# Cloud inversion analysis of surrounding rock parameters for underground powerhouse based on PSO-BP optimized neural network and web technology

**DOI:** 10.1038/s41598-024-65556-6

**Published:** 2024-06-22

**Authors:** Long Qu, Hong-Qiang Xie, Jian-Liang Pei, You-Gen Li, Jia-Ming Wu, Gan Feng, Ming-Li Xiao

**Affiliations:** 1 State Key Laboratory of Hydraulics and Mountain River Engineering, College of Water Resource and Hydropower, Sichuan University, Chengdu, 610065 China; 2 Sinohydro Bureau 7 Co., LTD, Chengdu, 610213 China

**Keywords:** Civil engineering, Power stations

## Abstract

Aiming at the shortcomings of the BP neural network in practical applications, such as easy to fall into local extremum and slow convergence speed, we optimized the initial weights and thresholds of the BP neural network using the particle swarm optimization (PSO). Additionally, cloud computing service, web technology, cloud database and numerical simulation were integrated to construct an intelligent feedback analysis cloud program for underground engineering safety monitoring based on the PSO-BP algorithm. The program could conveniently, quickly, and intelligently carry out numerical analysis of underground engineering and dynamic feedback analysis of surrounding rock parameters. The program was applied to the cloud inversion analysis of the surrounding rock parameters for the underground powerhouse of the Shuangjiangkou Hydropower Station. The calculated displacement simulated with the back-analyzed parameters matches the measured displacement very well. The posterior variance evaluation shows that the posterior error ratio is 0.045 and the small error probability is 0.999. The evaluation results indicate that the intelligent feedback analysis cloud program has high accuracy and can be applied to engineering practice.

## Introduction

Mechanical parameters of surrounding rock directly affect simulation results of underground powerhouse during excavation. Mechanical parameters used in numerical simulation are usually determined by in-situ or indoor mechanical experiments. However, sampling, testing, and human operations could influence experimental results. The obtained parameters may not accurately reflect the actual physical and mechanical properties of rock mass around the excavated cavern^1–4^. To solve this problem, in the 1970s, Kavanagh et al.^5^ first proposed the displacement back analysis method. The basic idea is to solve mechanical parameters of surrounding rock by the finite element method with actual monitoring displacements as the known quantity. Subsequently, the theory of back analysis has been continuously developed^6–8^. In recent years, with the development of artificial intelligence technology, many scholars have introduced intelligent methods such as the genetic programming (GP)^9–11^, the support vector machines (SVM)^12,13^, the artificial neural networks (ANN)^14^, and the particle swarm optimization (PSO)^15^ in inverse analyses of various typical engineering problems^16–20^. Various theoretical methods or optimization improvements have greatly improved the calculation speed and accuracy of the back analysis^21–25^.

For example, Feng et al.^26^ proposed a new displacement back analysis method by using the genetic algorithm (GA) to identify mechanical parameters of rock mass in large space search of highly non-linear multimodal. They used it to analyze the stability of the high side wall of the Three Gorges Project permanent shiplock. Feng et al.^27^ used the evolutionary support vector machine (ESVM) method for displacement inverse analysis of slopes based on the SVM and the GA. Zhao et al.^28^ proposed a new intelligent back analysis method by using the PSO algorithm to search the global optimization of the SVM, which can effectively identify geomechanical parameters. Majdi et al.^29^ studied the effectiveness of the GA in the structural design and optimization of the BP neural network and applied it to predict the deformation modulus of rock mass. Yang et al.^30^ combined a tension-free elastoplastic model with the GA optimization in back analysis to determine the rock parameters of nonlinear finite element simulations and to predict the behaviors of yield rock mass around deep tunnel. Gao et al.^31^ proposed a neural network inverse analysis method based on a black hole algorithm and analyzed the mechanical parameters of the surrounding rock of two deep roadways. The displacements calculated by the back-analyzed parameters agree with the measured values. Li et al.^32^ applied the PSO and the GA to the inverse analysis of the upper bound of finite element limit analysis. Zhang et al.^33^ developed an intelligent inverse analysis method combining the PSO and the GP machine learning with the finite difference method (FDM), and it has good global optimization capability and high computational efficiency. The above literature show that the inverse analysis methods based on intelligent algorithms were widely used in the inversion analyses of surrounding rock parameters in underground engineering^34^. Among these methods, the BP neural network is the most widely used method^35^. However, the BP neural network could quickly fall into the local extremum and has a slow convergence speed in practical applications. While, the PSO has superior performance in nonlinear function optimization and is suitable for optimizing the global optimal solution of complex space problems. Therefore, the PSO algorithm were adopted to optimize the initial weights and the thresholds of the BP neural network^36^.

Besides, it is worth noting that the parameters of surrounding rock will change with the development of excavation damage zone. Therefore, real-time inversion of surrounding rock parameters is needed to guide the construction of underground powerhouse^20,37^. To realize the real-time analysis of monitoring data and improve the utilization rate of monitoring data, cloud computing (CC)^38,39^ and internet of things (IOT)^40,41^ are gradually used in geotechnical engineering monitoring and analysis. For example, Sun et al.^42^ designed a tailings dam monitoring and pre-alarm system (TDMPAS) based on the IOT and the CC, which realized the real-time monitoring of the saturation line, water level, and dam deformation of a tailings dam. Rackwitz et al.^43^ developed a web-based client–server software platform to achieve data management in the area of geotechnical engineering risk and disaster prevention. Jiang et al.^44^ constructed a cloud service platform for dam safety monitoring based on a CC environment and a new monitoring model of dam safety. Yang et al.^45^ developed a monitoring and early warning system for underground engineering by integrating the IOT, CC, geotechnical simulation, artificial intelligence, and other technologies. Wang et al.^46^ also developed a monitoring and early warning cloud service platform for tunnel based on the IOT and CC. He et al.^47^ combined the IOT, CC, and big data technology to develop a multi-system and multi-parameter integrated monitoring and early warning system for rock burst and a remote monitoring cloud platform. To deal with a large number of monitoring data, cloud computing technology has the advantages of resource integration, data sharing, intelligent analysis, and fast calculation, and it creates some favorable conditions for real-time feedback analysis of surrounding rock parameters of underground powerhouse.

**Figure 1:**
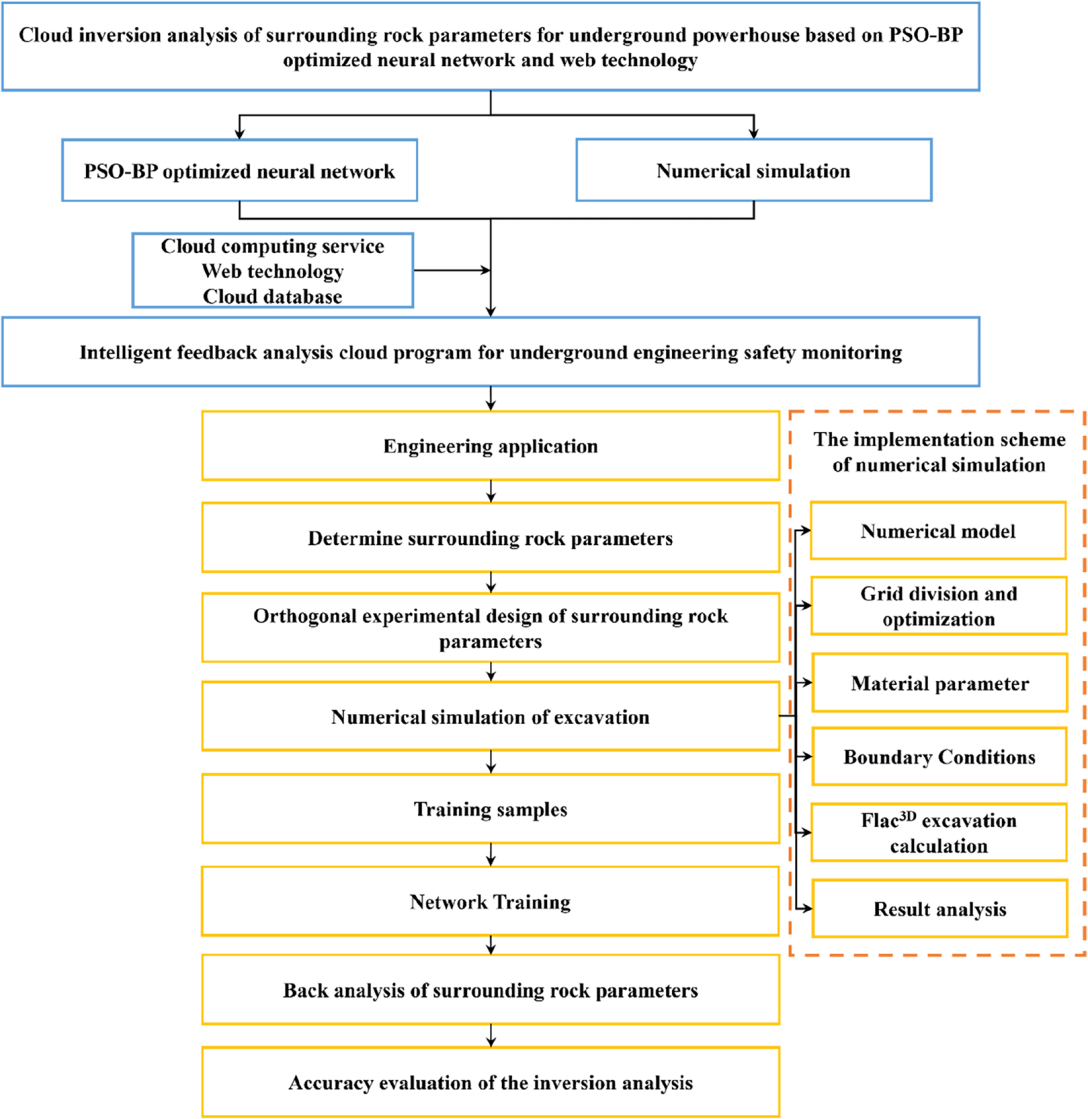
The structure of the study.

Therefore, the PSO-BP optimized neural network algorithm and numerical simulation were integrated into the cloud computing services to develop an intelligent feedback analysis cloud program for underground engineering safety monitoring in this study. The program can automatically perform the inversion analysis of surrounding rock parameters with some simple operations and quickly output the calculation results. The comprehensive structure of this study is shown in Fig. 1. Some basic algorithms of the cloud program were firstly introduced in this paper. Subsequently, the cloud program was applied to inversely analyze the surrounding rock parameters for the Shuangjiangkou underground powerhouse, which is under construction. Finally, the displacements simulated with the resulted surrounding rock parameters were compared with the monitoring data, and the accuracy of the program was evaluated by the posterior variance method.

## Intelligent feedback analysis cloud program based on the PSO-BP algorithm

### PSO-BP optimized neural network

BP neural network is a multi-layer feedforward network trained by data error reverse transfer algorithm^48^. Its network structure can contain multiple hidden layers. Theoretically, it can realize the nonlinear mapping from *n*-dimensional input space to *h*-dimensional output space^49^, so it could construct the nonlinear relationship between structural displacements and surrounding rock parameters.

**Figure 2:**
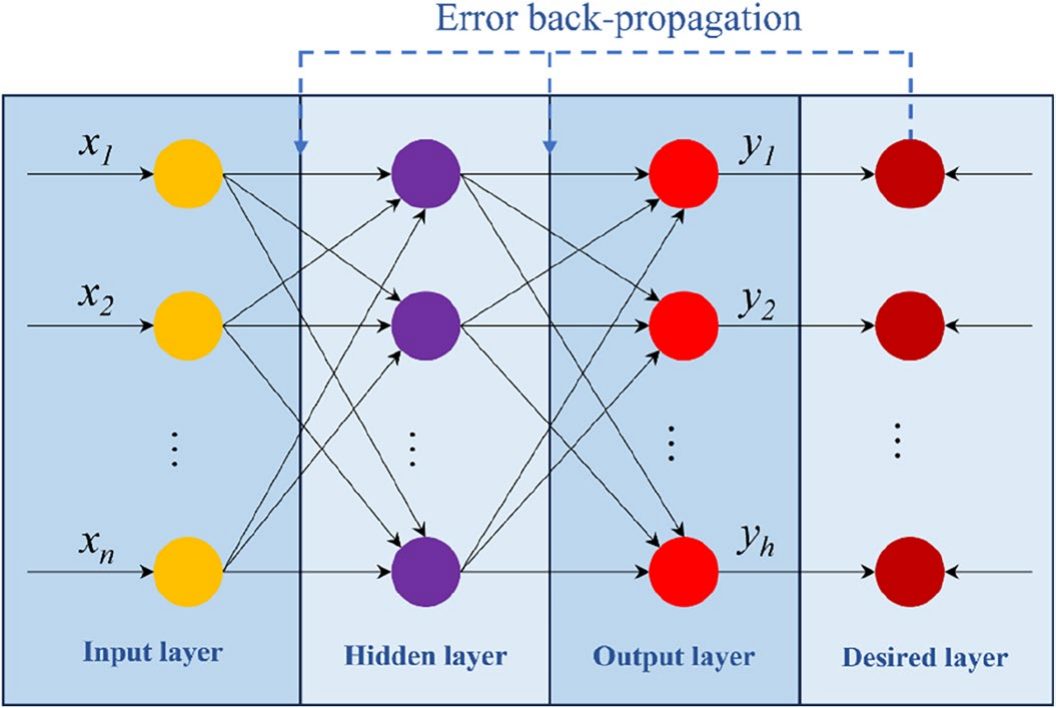
Topological structure of a BP neural network.

Figure 2 shows a typical topological structure of a BP neural network. Usually, a BP neural network consists of one input layer, one to multiple hidden layers, and one output layer. Each layer contains a number of nodes. When the number of input nodes is *n*, and the number of output nodes is *h*, the network is a mapping function from *n* independent variables to *h* dependent variables.

During the training process of the BP neural network, the deviation between the expected output and the actual output of the network keeps updating, and the weights and thresholds are adjusted automatically according to the deviation. Then, the deviation gradually decreases along the gradient direction until the accuracy requirement is met. The weights and thresholds that result in the convergency could be determined as a proper network for the parameter prediction of the surrounding rock. Therefore, the BP neural network can approach an unknown function with multiple independent and dependent variables through training.

Kennedy and Eberhart^50^ proposed the particle swarm optimization algorithm based on the principle of bird foraging, which is an effective global optimization algorithm. Particle swarm optimization (PSO) has the concept of population and evolution. The evolution of the population is the iterative calculation of the PSO algorithm. In each iteration, two extreme values are always concerned. One is the optimal position *p*_*best*_ (individual extreme value) of each particle in the iterative history, and the other is the optimal position *g*_*best*_ (global extreme value) of the whole particle swarm in the iterative history. After these two values are updated, the particles continuously change their velocity and position to obtain different fitness values. The fitness values obtained each time are compared with the fitness values of individual extremum and global extremum to obtain new individual extremum and fitness values. After cycles, the global optimal solution can be determined.

To improve the convergence performance of the PSO algorithm, Shi and Eberhart^51^ proposed a particle swarm optimization algorithm with an inertia weight factor *w*. In an *M*-dimensional target search space, for a group with *N* particles, when the *t*-th iteration obtains the optimal position, the position and velocity of a particle *i* in the *j*-dimensional component can be updated as^19,33,51^:

1 $$ v_{ij} (t + 1) = wv_{ij} (t) + c_{1} r_{1} (t)\left[ {p_{ij} (t) - x_{ij} (t)} \right] + c_{2} r_{2} (t)\left[ {p_{gj} (t) - x_{ij} (t)} \right] $$

2 $$ x_{ij} (t + 1) = x_{ij} (t) + v_{ij} (t + 1) $$

where *x*_*ij*_ and *v*_*ij*_ are the position and velocity of the *i*th particle on the *j*th dimensional component, respectively; *p*_*best_ij*_ and *g*_*best_ij*_ are the individual extremum and the global extremum of the *i*th particle on the *j*th dimensional component, respectively; *c*_1_ and *c*_2_ are acceleration constants, generally *c*_1_ = *c*_2_ = 1.5–2.5; *r*_1_ and *r*_2_ are random numbers of 0 ~ 1; *w* is called the inertia weight, and the value of *w* has a significant influence on the optimization ability of particle swarm optimization. If the value of *w* is large, the algorithm has a strong ability in global optimization. If the value of *w* is small, the algorithm has a strong ability in local search. In the iterative process, the inertia weight *w* follows the linear attenuation weight strategy^33,51^:

3 $$ w = w_{\max } - \frac{{w_{\max } - w_{\min } }}{{t_{\max } }}t $$

where *t* is the current iteration number; and *t*_max_ is the maximum number of iterations; in general, *w*_min_ = 0.8 and *w*_max_ = 1.2.

**Figure 3:**
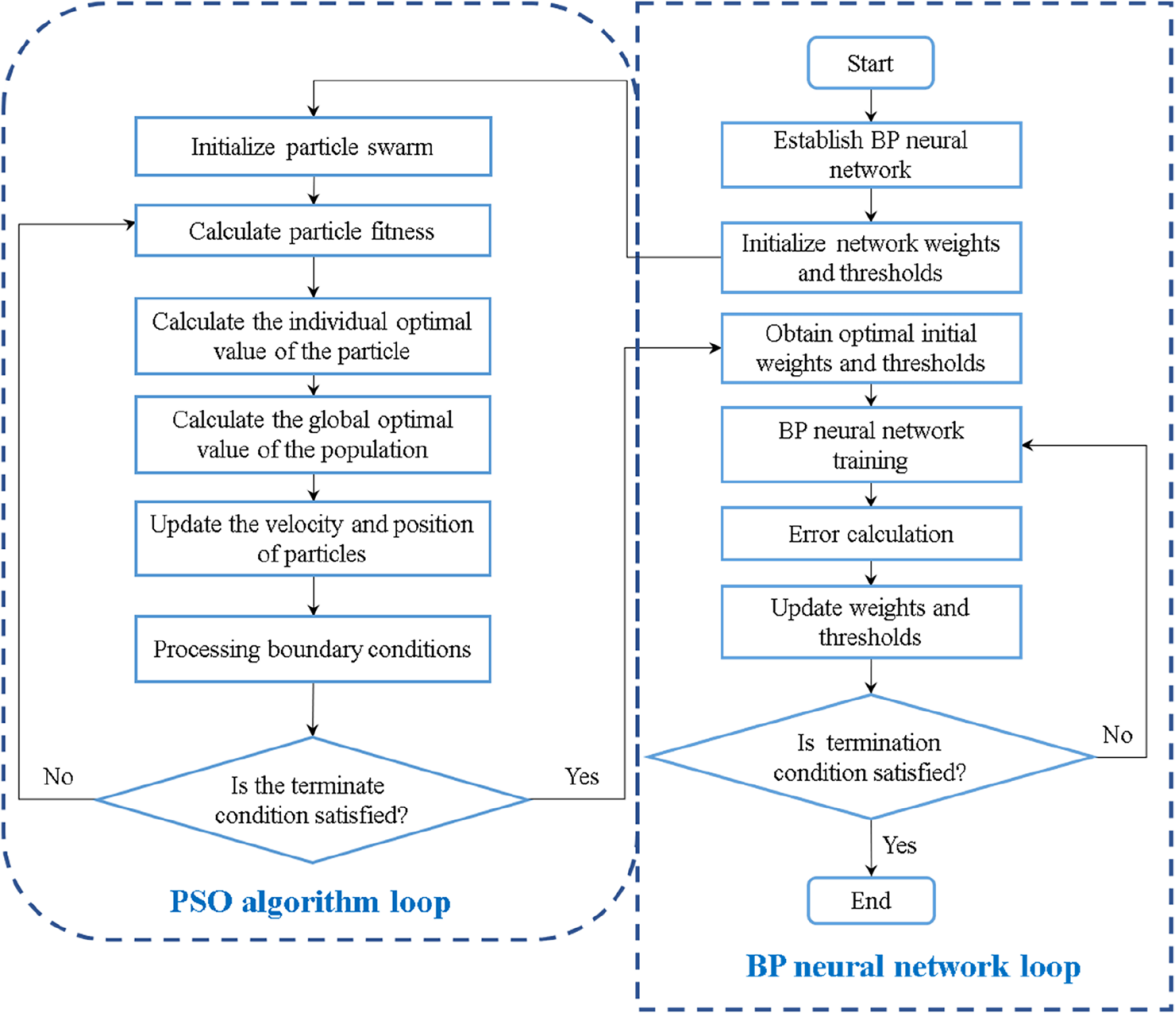
Flow chart of PSO-BP neural network.

In the practical application of solving complex nonlinear problems, the initial parameters of the BP neural network structure are difficult to determine. On the one hand, the BP neural network needs to initialize many parameters, such as network layers, nodes, weights, thresholds, iterations, and error tolerance. These parameters can only be given roughly according to experience, which may not be suitable for a specific problem. On the other hand, the BP neural network is susceptible to the initial weights. A small change of that will lead to fall into local extremum. The reason is that the BP neural network is based on the gradient descent. From a mathematical perspective, there must be local minima when using the gradient descent method to solve the minimum problem, and the algorithm is prone to falling into local minima. It cannot obtain the global optimal solution. The particle swarm optimization is a global algorithm and does not have local convergence issues. Therefore, the optimization ability of the PSO algorithm is used to optimize the initial weights and thresholds of the BP neural network, to improve the speed of convergence and the accuracy of results. The process of optimizing the BP neural network by the PSO is shown in Fig. 3. The specific implementation steps are as follows:

1. Establish the BP neural network structure, and set the number of nodes in the input layer, hidden layer, output layer, and other initial network parameters.
2. Initiate particle swarm parameters, such as population size *N*, particle position *x*_*i*_ and velocity *v*_*i*_, inertia weight *w*, acceleration constants *c*_1_ and *c*_2_, maximum velocity *v*_max_, and maximum number of iterations; the dimension *M* of the particle is the sum of all weights and thresholds of neural network. If the numbers of nodes in the input layer, hidden layer, and output layer of a three-layer BP network structure are *n*, *m*, and *h*, respectively, the dimension of the particle swarm is:
  4 $$ M = n \times m + m + m \times h + h $$
3. The mean square error (MSE) of the neural network is used as the fitness function to calculate the fitness value *fitness*[i] of all particles in each iteration.
4. Update the individual extremum. For each particle, if the fitness value *fitness*[i] < the individual extremum *p*_*best*_, then $p_{best} = fitness[{\text{i}}]$.
5. Update the global extremum. For each particle, if the fitness value *fitness*[i] < global extremum *g*_*best*_, then $g_{best} = fitness[{\text{i}}]$.
6. Iteratively update the particle position *x*_*i*_ and velocity *v*_*i*_, and ensure that the velocity and position do not exceed the limited range.
7. Termination conditions. If the maximum number of iterations is completed or the *fitness*[i] meets the accuracy requirements, the iteration is terminated. Then, the optimal result is output. Otherwise, return to step (3).

### Intelligent feedback analysis cloud program for underground engineering safety monitoring

The PSO-BP optimized neural network, numerical simulation, cloud computing services, web technology, and cloud database were integrated to construct an intelligent feedback analysis cloud program for underground engineering safety monitoring. The program can perform intelligent real-time feedback cloud analysis through cloud computing services. The user only needs to log on to the web page platform and execute some simple operations, and the program automatically performs simulation calculations and quickly output inversion analysis results.

**Figure 4:**
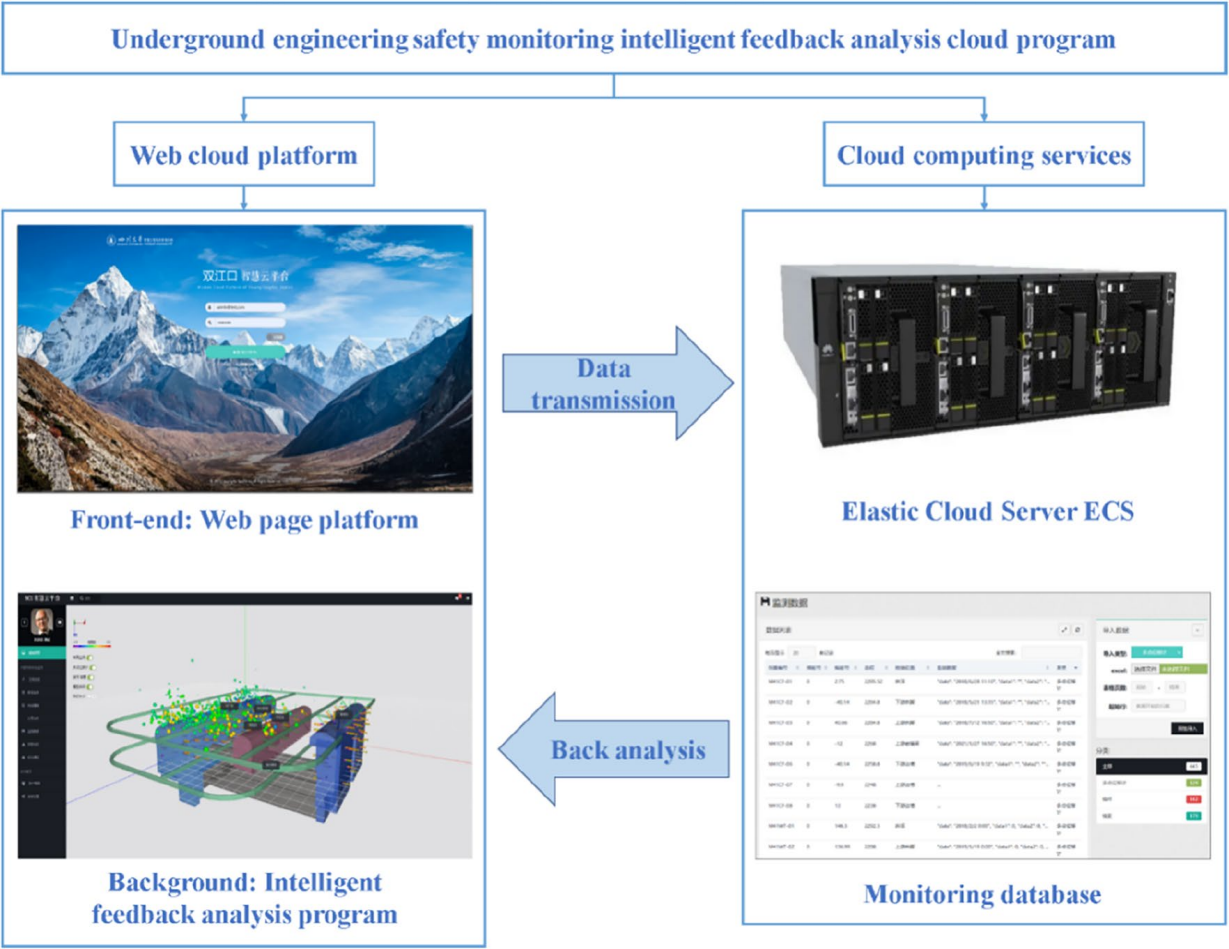
Basic structure of intelligent feedback analysis cloud program for underground engineering safety monitoring.

The basic structure of the program is shown in Fig. 4. In this paper, the web cloud platform includes a web client (front-end) and a web server (background)^52^. The front-end is a direct object for users to manage monitoring data and control numerical calculation, while the background is to complete data storage, software invocation, numerical simulation, and parameter inversion. Web application development is mainly based on Browser/Server (B/S) structure mode. The front-end collects data input by user and sends it to the server, and the server responds to the front-end after completing data processing. In this program, the front-end is developed based on the Bootstrap framework, and the background server is developed by Hypertext Preprocessor (PHP). The front-end is deployed on the cloud server. Moreover, the cloud computing service is on the Huawei Elastic Cloud Server (ECS), and the GaussDB (for MySQL) cloud database is built on the ECS to store the monitoring data.

**Figure 5:**
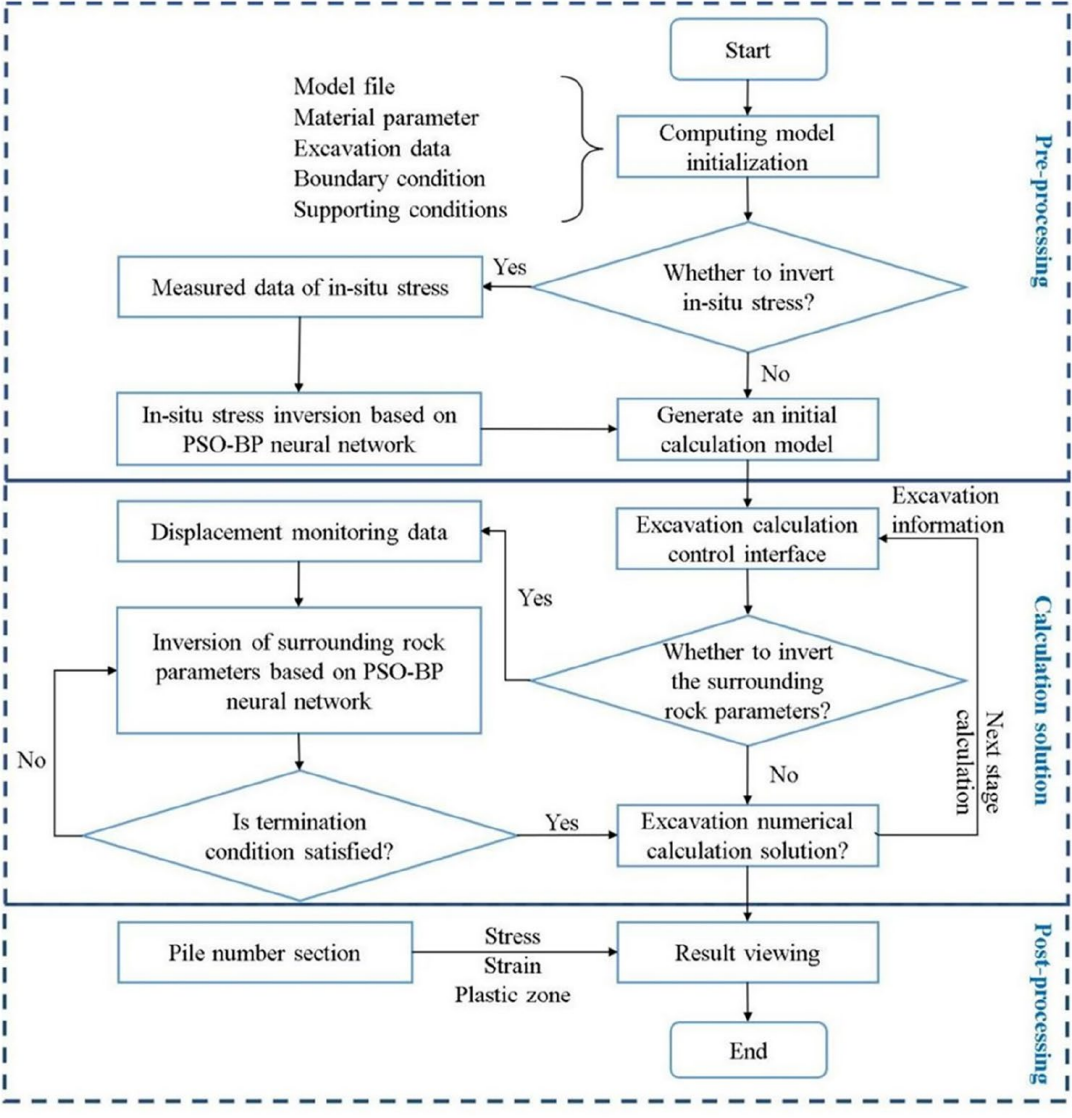
Flow chart of intelligent back analysis.

Multiple languages, such as the Fish language of FLAC^3D^, MATLAB, Python and PHP, were used to systematically develop the intelligent feedback analysis program for calling software, executing commands, and exchanging data in the background of the server. The remote interface control of the intelligent feedback analysis program was realized through the web platform on the front-end. The back analysis process of the program can be divided into three stages, including pre-processing, calculating, and post-processing, as shown in Fig. 5.

**Figure 6:**
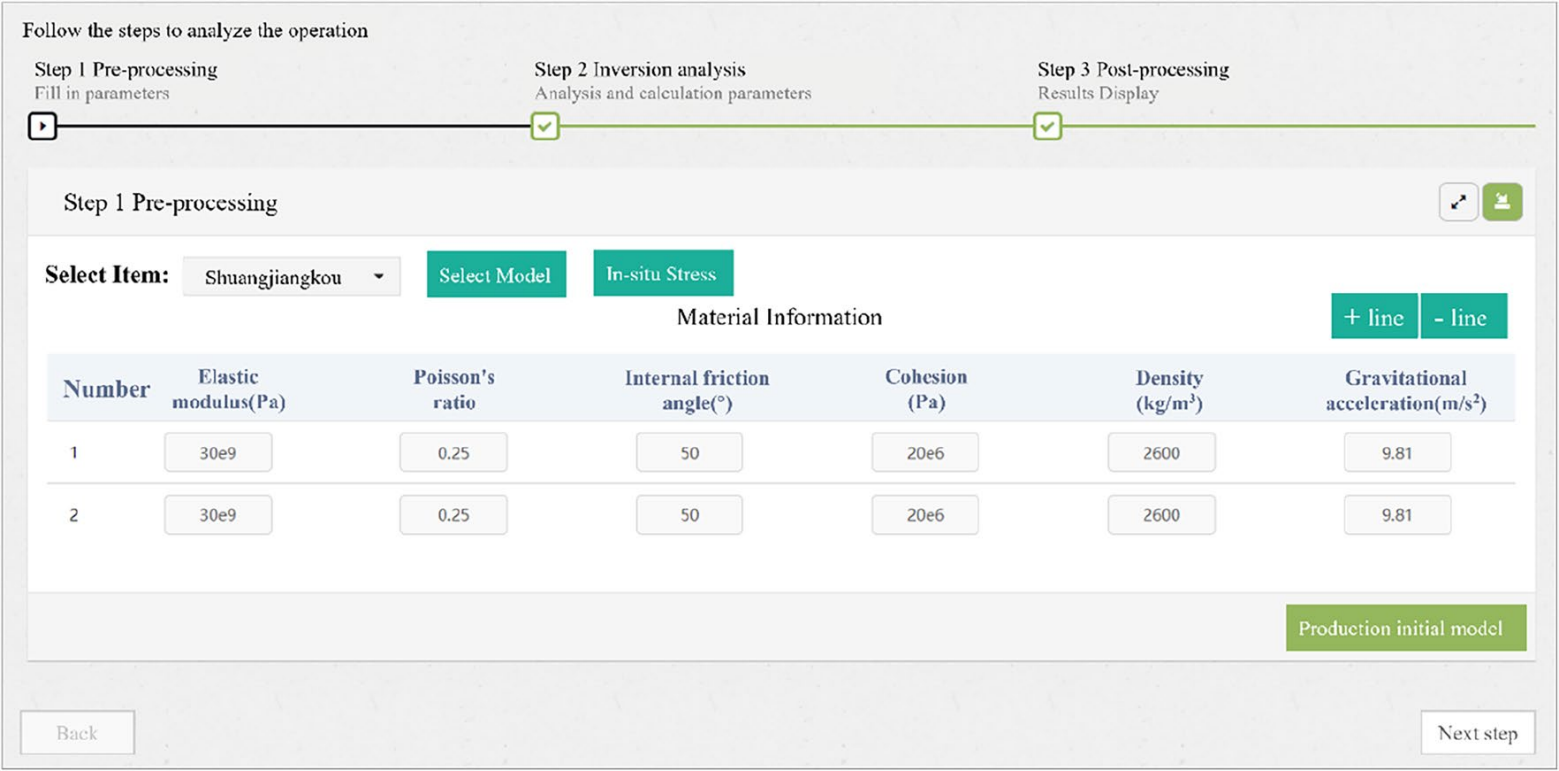
Pre-processing control interface on the web platform.

**Figure 7:**
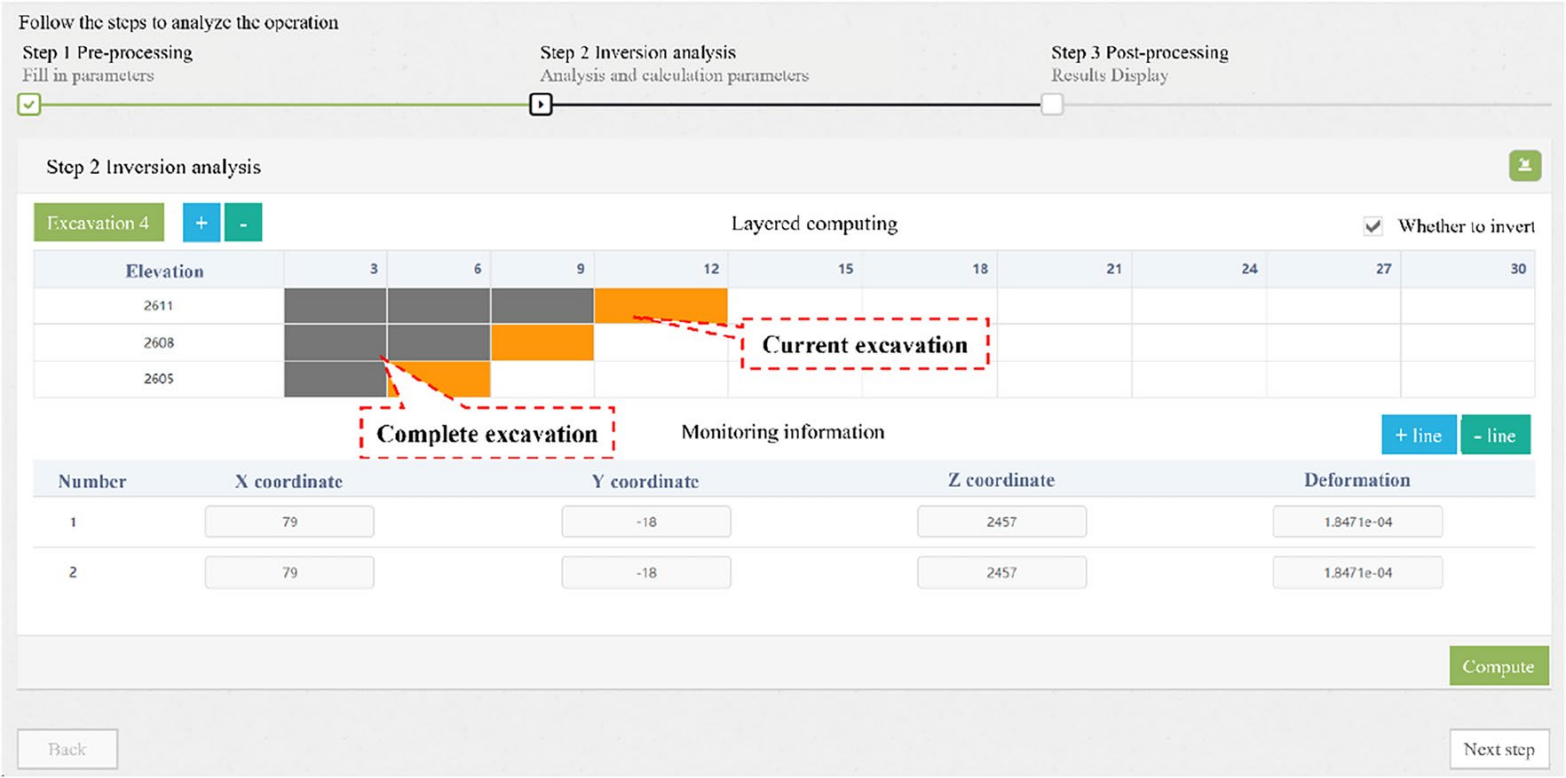
Computing control interface on the web platform.

**Figure 8:**
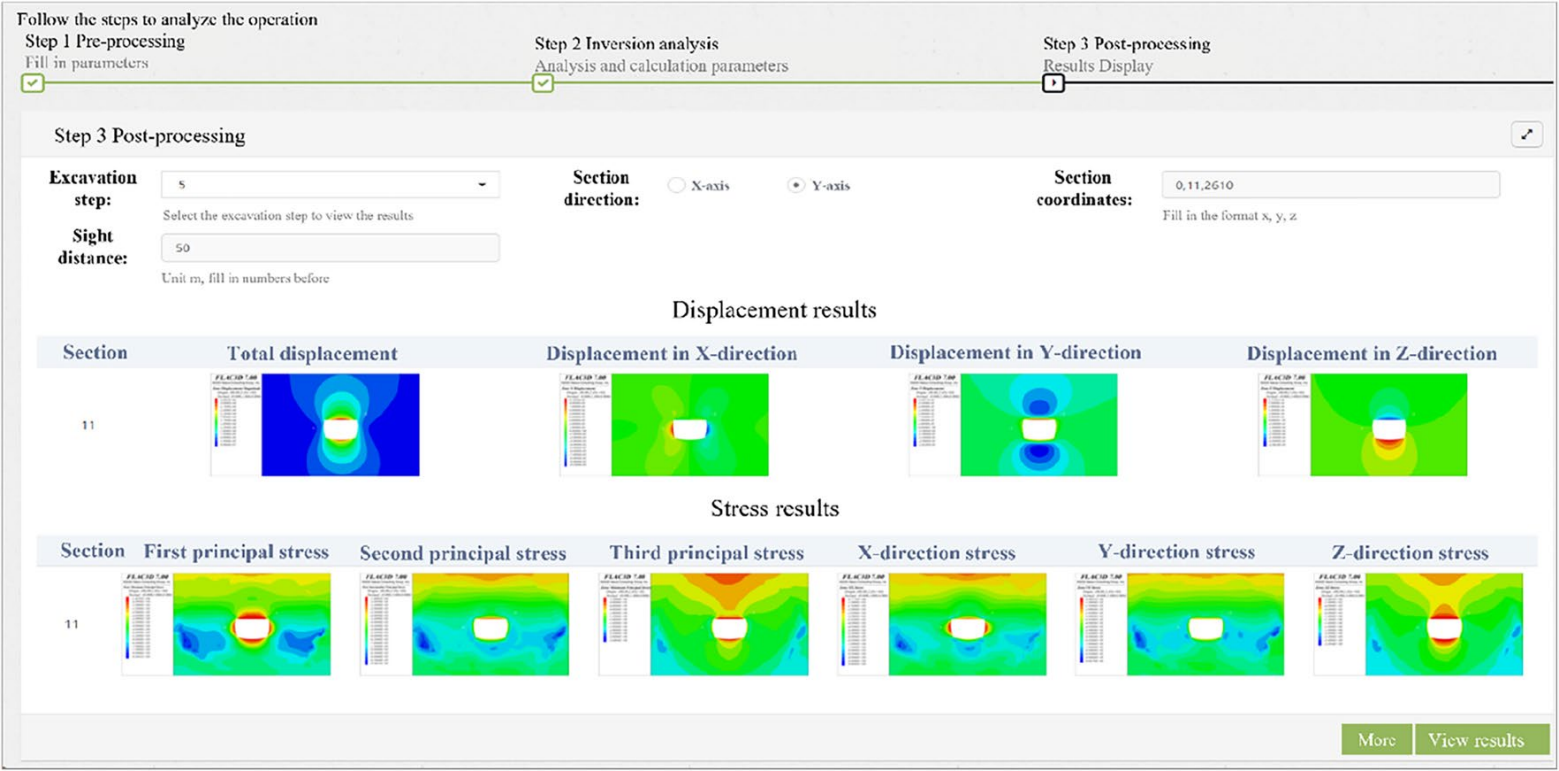
Post-processing control interface on the web platform.

1. The pre-processing includes model initialization and optional in-situ stress inversion, which could also be inversely analyzed by the PSO-BP algorithm and has already been investigated by the authors’ coworker^53^. The items and models saved in the database should be selected in the pre-processing. After the initial parameters, such as material parameters and measured in-situ stress, are input, the initial model can be generated. If the measured in-situ stress is input, the program will perform the in-situ stress inversion automatically. Otherwise, the initial model is only subjected to gravity. Subsequently, FLAC^3D^ is called to generate displacement boundaries and initial stress field for the numerical model, which will used for the following excavation simulation and inversion analysis of surrounding rock parameter. The pre-processing interface is shown in Fig. 6.
2. The calculating process includes excavation simulations to generate training samples and inversed analysis of surrounding rock parameters. Firstly, the computing control interface shown in Fig. 7 is generated based on the excavation information uploaded to the cloud database. Secondly, the user must assign the zones that need to be excavated for every step. Figure 7 shows a three-bench excavation process of a tunnel with a footage of 3 m. The gray fillings represent the excavated parts, and the orange fillings represent the parts that will be excavated in current excavation step. The rock to be excavated is selected for each calculation step. By clicking the "compute" button, the program will call the FLAC^3D^ to complete the excavation simulation and return the results. Thirdly, before sending the simulation instruction, user can choose whether to inversely analyze the surrounding rock parameters for the current excavation step. If inversion analysis is not selected, the program will simulate the excavation using the initial parameters of the surrounding rock. If inversion analysis is selected, the displacements and coordinates of the monitoring points should be input or automatically called from monitoring database if pre-defined, and the program of the PSO-BP optimized neural network will be executed for parameter inversion analysis. The procedure of the inversion analysis can be summarized as follows. The program generates a series of orthogonal experiments based on the initial parameters of the surrounding rock and runs these numerical simulations automatically to obtain training samples. In addition, the program for the inversion analysis is called in the background to train the network and establish a nonlinear relationship between the displacements and the parameters of surrounding rock. Finally, the program predicts the surrounding rock parameters by substituting the monitored displacements into the trained network. Based on the back-analyzed parameters, the program simulates the excavation again for the current excavation step as the final result.
3. The post-processing includes the visualization of the results. The concerned excavation steps could be selected on the web platform, and the control data of visualization, such as section direction and coordinates, should be input. The program will quickly generate the displacement and stress distribution and return them to the web platform. For example, Fig. 8 shows the displacement and stress distribution of a cavern after the fifth step of excavation. In addition, more visions can be generated by clicking the "more" button, such as the back-analyzed parameters of surrounding rock, the plastic zone distribution and the simulated and monitoring displacement or stress curves of the monitoring points.

## Engineering application of the PSO-BP intelligent feedback analysis cloud program

### Engineering overview

The Shuangjiangkou Hydropower Station is located in Jinchuan County, Aba Tibetan and Qiang Autonomous Prefecture, Sichuan Province, China. The normal storage elevation of the reservoir is 2500 m, the total storage capacity is 2.897 billion m^3^, and the regulating storage capacity is 1.917 billion m^3^. The installed capacity of the power station is 2000 MW, and the average annual power generation is 7.707 billion kW·h.

The surrounding rock of the underground powerhouse mainly consists of porphyritic biotite K-feldspar granite. A lamprophyre vein is developed around the underground powerhouse. Its width is about 0.8–1 m, and its surfaces are staggered and eroded into a 3–5 cm clastic rock. The fault numbered as F1 is developed around the underground powerhouse, and it has an occurrence of N79°W/SW∠48° and a crushing bandwidth of 50–60 cm. It is comprised of clastic rocks and contains a layer of secondary mud, whose thickness is about 1–4 mm. Dripping water could fall from the fault F1. The surrounding rock has a good integrity, and its structure presents as a large block structure. The wall of the exploration tunnel is dry, and seepage, drip water and slight flowing water were observed in a few tunnel sections. Thus, most of the surrounding rock is classified as class IIIa, and the surrounding rock near the faults and lamprophyre veins are classified as class IV–V in the geological survey report of the Shuangjiangkou Hydropower Station. The quality of rock mass is generally good for the construction of the underground powerhouse. The maximum principal stress has a small angle with the axis of the underground powerhouse, which is beneficial to the overall stability of the cavern. However, its top arch, side walls, and end walls are affected by the lamprophyre veins and different structural planes, and the local stability is poor around these unfavorable geological structures.

### Excavation and support scheme of underground powerhouse

**Figure 9:**
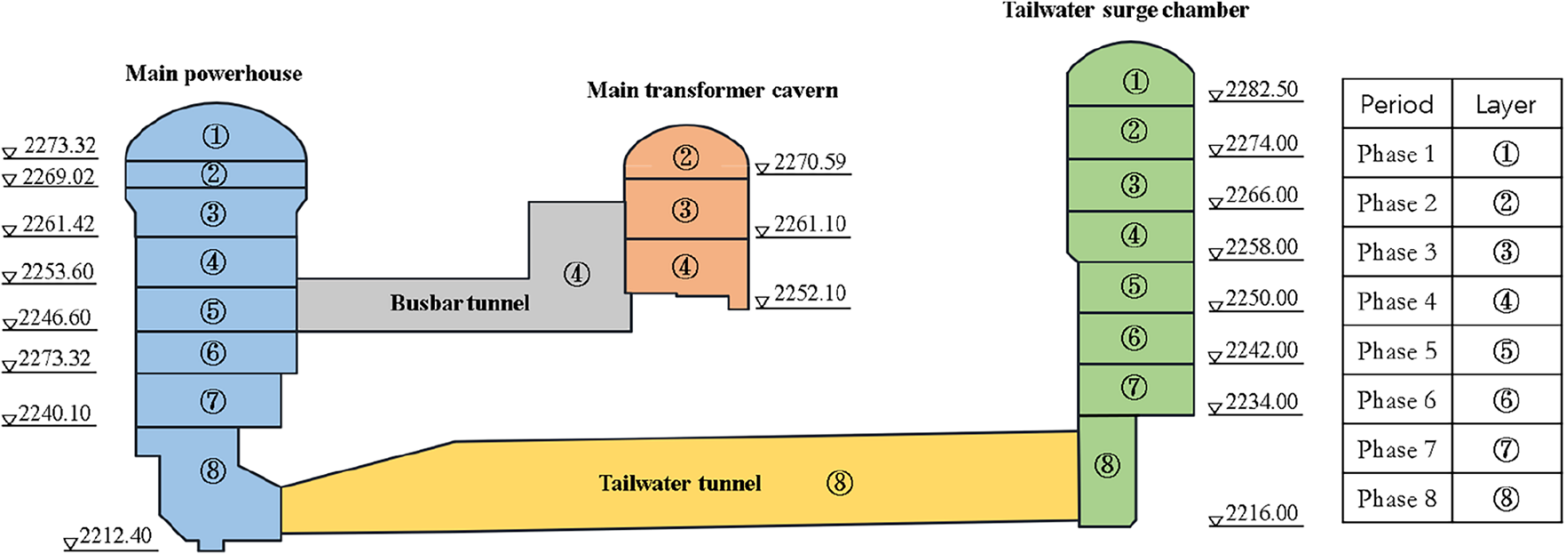
Excavation scheme of the Shuangjiangkou underground powerhouse.

The excavation scheme of the Shuangjiangkou underground powerhouse is shown in Fig. 9. The excavation of the underground powerhouse is mainly divided into eight construction periods. The three major caverns are excavated in parallel during the corresponding construction period. As of March 2022, the Shuangjiangkou underground powerhouse has been excavated to the fourth layer. The multi-point displacement meters were installed after the surrounding rock at the installation position was exposed. After the first layer of excavation, only several multi-point displacement meters were installed, so the monitoring results of the first layer cannot represent the deformation of the completely excavated powerhouse and are not suitable for inversion analysis. After the fourth layer of excavation, the excavated underground cavern is large enough to reveal the properties of surrounding rock. Therefore, the monitoring data from the initial four stages of excavation were chosen to inversely analyze the mechanical parameters of surrounding rock.

**Figure 10:**
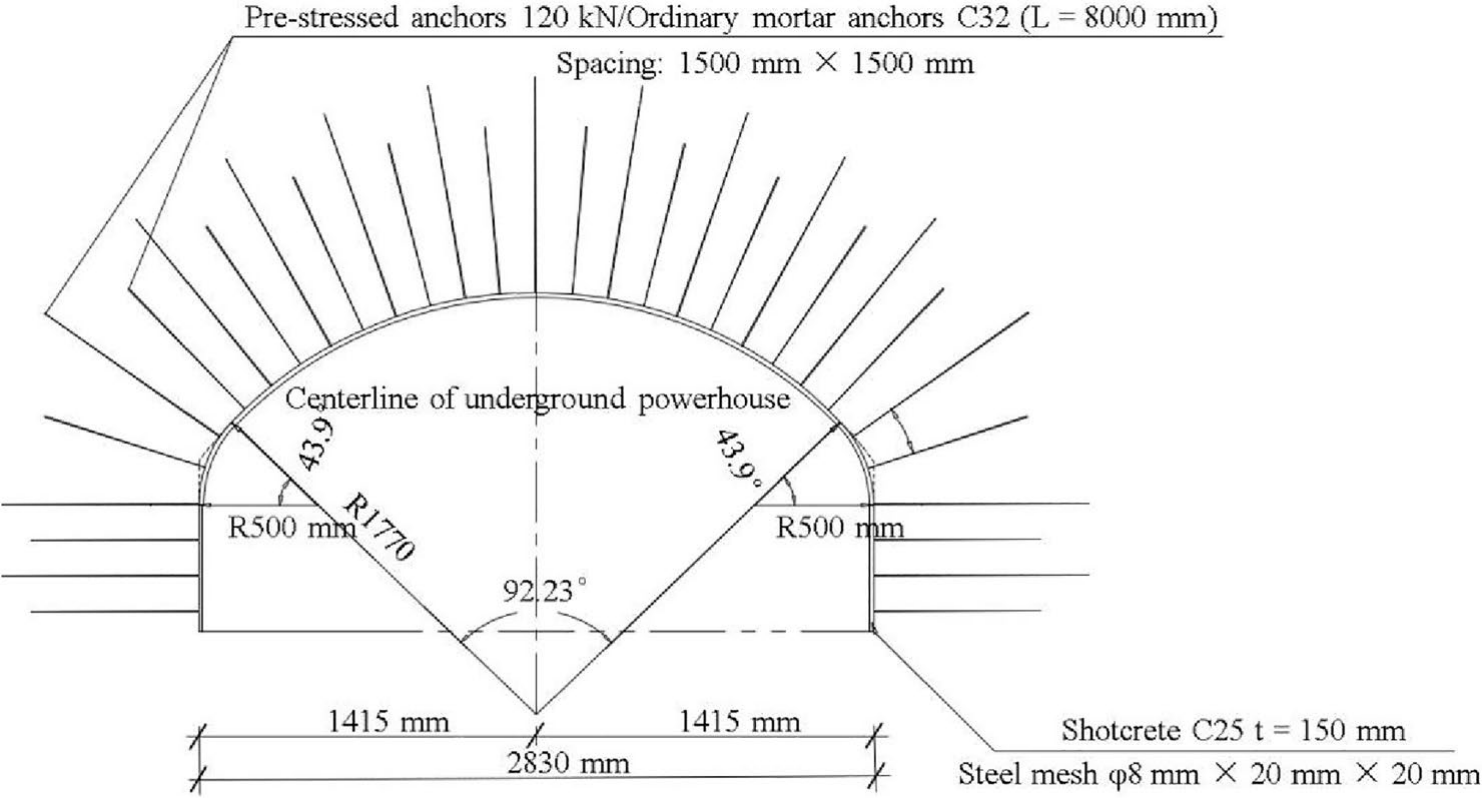
Supporting scheme of the Shuangjiangkou underground powerhouse.

The supporting structures of the underground powerhouse include shotcrete, anchors and steel meshes. The shotcrete thickness is 15 cm, and its concrete grade is C25. Two kinds of anchors including ordinary mortar anchors and pre-stressed anchors were used to support the vault, side wall, and end wall of the underground powerhouse, and the anchors have a spacing of 1.5 m × 1.5 m. The grade of ordinary mortar used for anchors is M30. The support scheme of the Shuangjiangkou underground powerhouse is shown in Fig. 10.

### Numerical model

According to the design of the Shuangjiangkou underground powerhouse, the model includes the terrain and stratum, the faults, the lamprophyre vein, the cavern structure of the powerhouse, and the anchors. The plane range of the model is in a 700 m × 700 m square area. The bottom elevation of the model is 2000 m, and its top extends to the ground surface.

**Figure 11:**
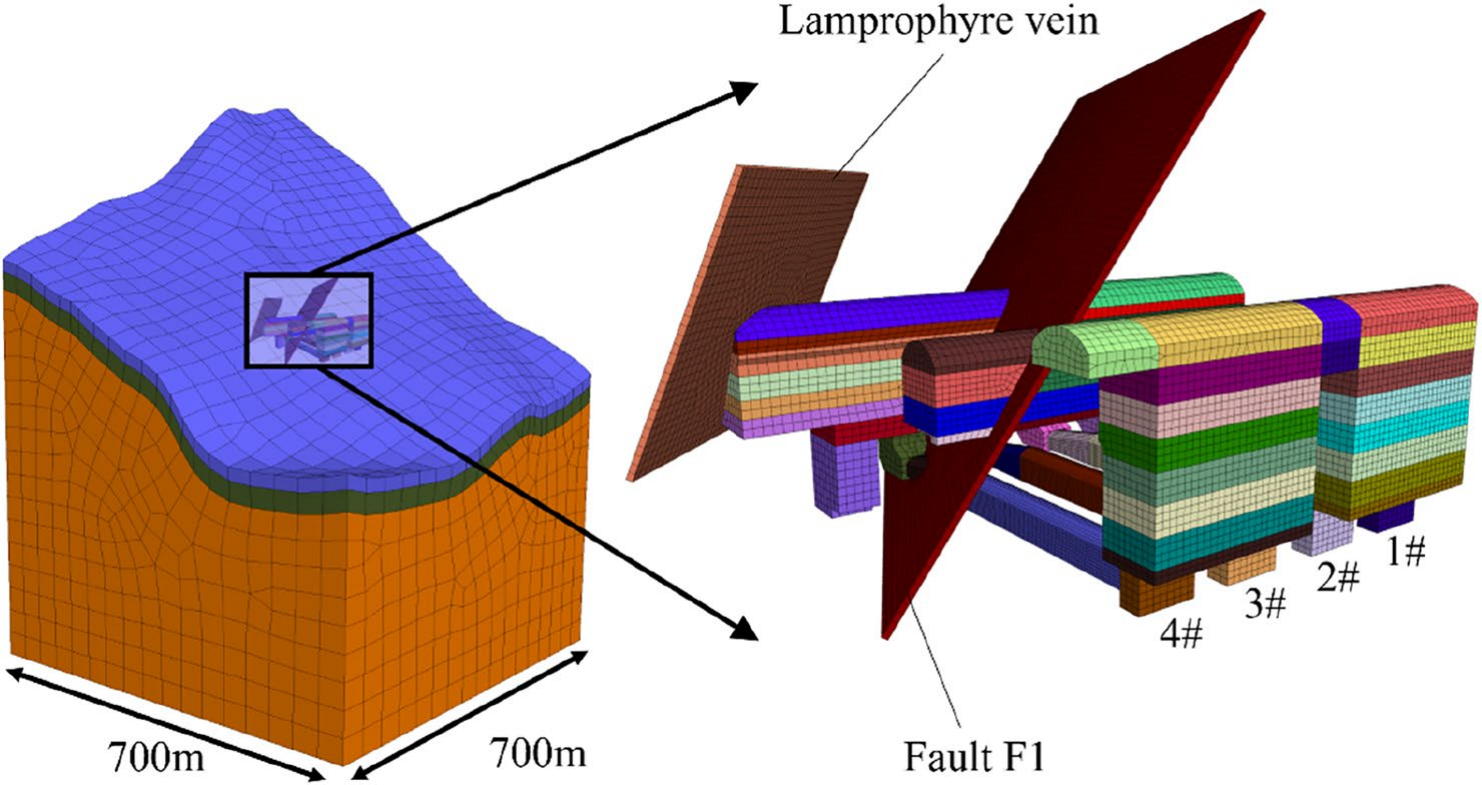
Numerical model of the Shuangjiangkou underground powerhouse.

To optimize the mesh, using one-tenth of the minimum edge length of the underground cavern as the mesh edge length not only ensures the precision of the mesh but also does not cause excessive mesh quantity. The numerical model imported into FLAC^3D^ is shown in Fig. 11. A total of 477,685 nodes and 753,151 elements are meshed in the model. The elements are mainly hexahedral elements with eight-node, and their shapes are good. The strength criterion follows the Mohr–Coulomb criterion. The unbalanced force of convergence is set to the default value of 10^−5^. The link elements were used to simulate the ordinary mortar anchors and the pre-stressed anchors.

Based on the in-situ stress measurement results around the Shuangjiangkou underground powerhouse, the initial in-situ stress field and boundary conditions of this numerical model has been inversely analyzed by the authors’ coworkers^53^. Their work on the back analysis of the in-situ stress is also an important work integrated in the cloud program, so their analysis results of the in-situ stress were directly used in the back analysis of surrounding rock parameters. Initially, normal displacement constraints were applied on the bottom, left, and back sides of the model, and the back-analyzed stress conditions were applied on their corresponding boundaries.

The geological conditions of the studied area include fresh granite, weakly weathered granite, strongly weathered granite, lamprophyre vein, and fault. Before the simulation, their mechanical and other necessary parameters were input on the web platform. These parameters were obtained from the geological survey report of the Shuangjiangkou Hydropower Station and are listed in Table 1.

**Table 1:** The mechanical parameters of numerical model.

Type of rock mass	Density/kg/m^3^	Young's modulus *E*/GPa	Poisson's ratio *μ*	Cohesion *c*/MPa	Friction coefficient f
Fresh granite	2600	32.9	0.25	1.5	1.3
Weakly weathered granite	2550	25.0	0.30	1.0	1.0
Strongly weathered granite	2350	20.0	0.32	0.5	0.8
Lamprophyre vein	2300	6.0	0.35	0.1	0.35
Fault	2300	6.0	0.35	0.1	0.35
Steel	7890	209	0.27	\	\

### Training samples

**Figure 12:**
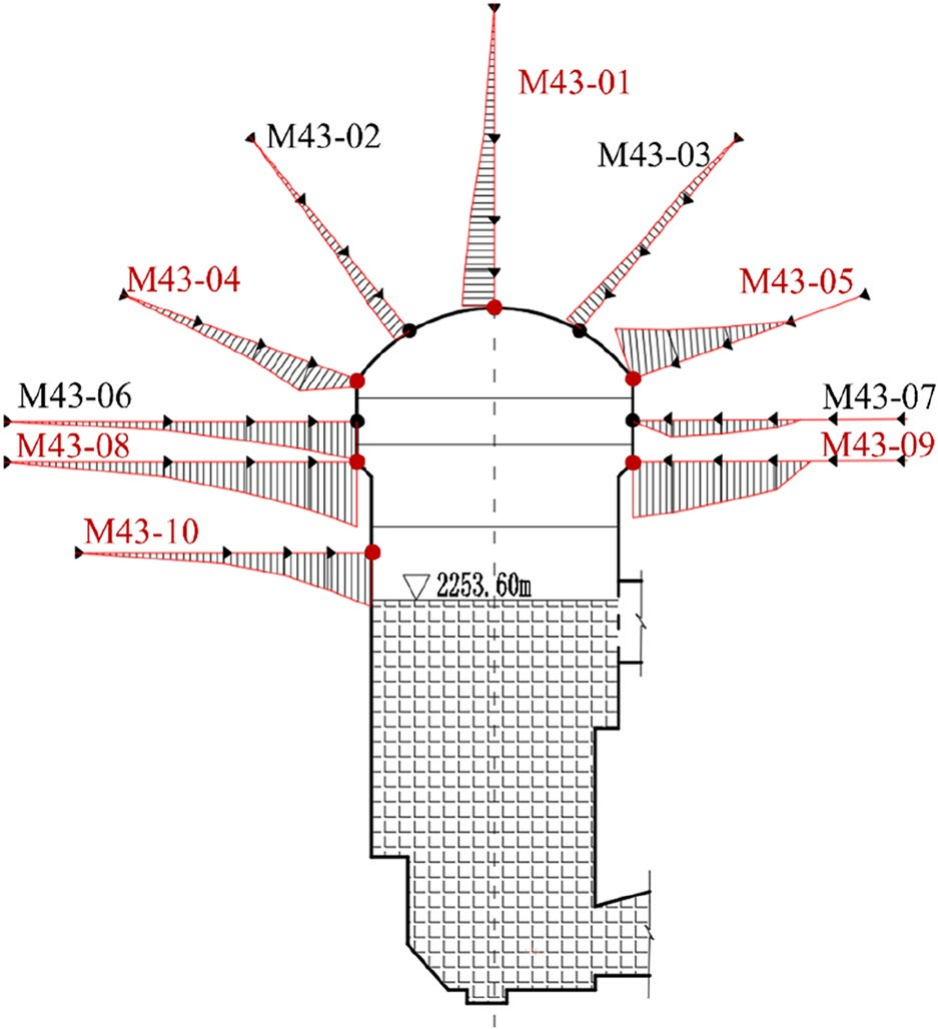
Installation position of the multi-point displacement meters in the section of 3# electric generator.

The PSO-BP back analysis method was used to analyze the parameters of the surrounding rock. The fault F1 crosses the main powerhouse around the 3# electric generator, so the cross section of the 3# electric generator was chosen as the characteristic section to conduct the back analysis. As shown in Fig. 12, the section contains 10 multi-point displacement meters, which record the displacement of 10 points on the surface of the excavated cavern. The monitoring data measured from only six of them (marked in red) are used for back analysis to control the number of samples. These six multi-point displacement meters are chosen alternately to keep the accuracy, as listed in Table 2. Besides, the displacement increments of these six meters during the fourth layer of excavation are taken as the target of the back analysis.

**Table 2:** Measured displacement increments of five monitoring points during the fourth layer of excavation.

Number of monitoring point	Location	Displacement increment/mm
M43-01	Vault	0.33
M43-04	Upstream arch	1.57
M43-05	Downstream arch	1.96
M43-08	Upstream rock anchor beam	4.53
M63-09	Downstream rock anchor beam	5.61
M43-10	Upstream side wall	5.83

The surrounding rock parameters considered in the back analysis include the deformation parameters and the Mohr–Coulomb strength parameters, i.e., modulus *E*, Poisson's ratio *μ*, internal friction angle *φ*, and cohesion *c*. The initial parameters of surrounding rock are obtained from the geological survey report of the Shuangjiangkou Hydropower Station, as listed in Table 3.

**Table 3:** Initial parameters of surrounding rock of the Shuangjiangkou powerhouse.

Parameter	Modulus *E*/GPa	Poisson 's ratio *μ*	Internal friction angle *φ*/°	Cohesion *c*/MPa
Reference value	32.9	0.25	52.4	1.5

According to the principle of orthogonal design^54,55^, the web cloud platform was used to carry out the orthogonal experiments to obtain some sample data. There were four mechanical parameters of surrounding rock in the back analysis, and seven values of each parameter were designed, i.e., 0.7, 0.8, 0.9, 1.0, 1.1, 1.2, and 1.3 times of its initial value listed in Table 3. Thus, the orthogonal test scheme table was designed as ${\text{L}}_{49} (7^{4} )$, which was integrated into the intelligent feedback analysis cloud program. After inputting the initial surrounding rock parameters on the web platform, the program automatically generated an orthogonal test plan, as listed in Table 4.

**Table 4:** Orthogonal test scheme of rock parameters.

Test number	*E*/GPa	*μ*	*φ*/°	*c*/MPa	Test number	*E*/GPa	*μ*	*φ*/°	*c*/MPa
1	24.50	0.18	35.70	0.91	26	24.50	0.20	45.90	1.04
2	38.50	0.30	66.30	1.04	27	38.50	0.33	40.80	1.17
3	28.00	0.25	61.20	1.17	28	28.00	0.28	35.70	1.30
4	42.00	0.20	56.10	1.30	29	31.50	0.30	40.80	1.30
5	31.50	0.33	51.00	1.43	30	45.50	0.25	35.70	1.43
6	45.50	0.28	45.90	1.56	31	35.00	0.20	66.30	1.56
7	35.00	0.23	40.80	1.69	32	24.50	0.33	61.20	1.69
8	38.50	0.25	45.90	1.69	33	38.50	0.28	56.10	0.91
9	28.00	0.20	40.80	0.91	34	28.00	0.23	51.00	1.04
10	42.00	0.33	35.70	1.04	35	42.00	0.18	45.90	1.17
11	31.50	0.28	66.30	1.17	36	45.50	0.20	51.00	1.17
12	45.50	0.23	61.20	1.30	37	35.00	0.33	45.90	1.30
13	35.00	0.18	56.10	1.43	38	24.50	0.28	40.80	1.43
14	24.50	0.30	51.00	1.56	39	38.50	0.23	35.70	1.56
15	28.00	0.33	56.10	1.56	40	28.00	0.18	66.30	1.69
16	42.00	0.28	51.00	1.69	41	42.00	0.30	61.20	0.91
17	31.50	0.23	45.90	0.91	42	31.50	0.25	56.10	1.04
18	45.50	0.18	40.80	1.04	43	35.00	0.28	61.20	1.04
19	35.00	0.30	35.70	1.17	44	24.50	0.23	56.10	1.17
20	24.50	0.25	66.30	1.30	45	38.50	0.18	51.00	1.30
21	38.50	0.20	61.20	1.43	46	28.00	0.30	45.90	1.43
22	42.00	0.23	66.30	1.43	47	42.00	0.25	40.80	1.56
23	31.50	0.18	61.20	1.56	48	31.50	0.20	35.70	1.69
24	45.50	0.30	56.10	1.69	49	45.50	0.33	66.30	0.91
25	35.00	0.25	51.00	0.91					

### Network training

The program called FLAC^3D^ in the server to carry out 49 groups of numerical simulations to obtain the displacement increment of surrounding rock during the fourth layer of excavation. It outputted the displacement increments of six monitoring points listed in Table 2. After the numerical simulations, the Matlab was automatically called for network training.

**Figure 13:**
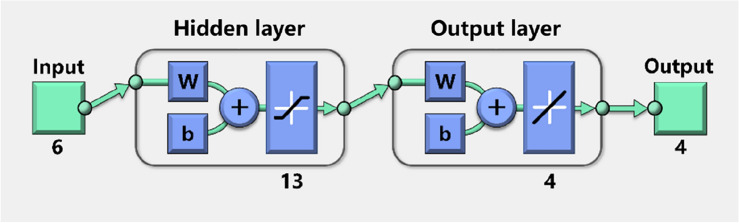
PSO-BP neural network structure.

The simulated displacement increments of six monitoring point were taken as the input samples, and the surrounding rock parameters of each numerical simulation were taken as the output samples. The PSO-BP network were trained by these input and output samples. The parameters of the neural network were initialized as follows. The number of nodes in the hidden layer is 13, and the transfer function is tansig. The number of the output layer is 4, and the transfer function is purelin. Therefore, a 6-13-4 topology network were preset in the program, as shown in Fig. 13. The network function of error backpropagation was selected as the newff function, and the training function was selected as the trainscg function. The maximum number of network training was set to 300,000, the learning rate was set to 0.001, and the target tolerance was set to 10^−6^.

The parameters of the PSO were initialized as follows. The group size *N* was set to 60, the maximum number of iterations *t*_max_ was set to 300, the maximum particle velocity *v*_max_ was set to 0.5, the particle acceleration constants *c*_1_ and *c*_2_ were set to 2, the inertia weight was taken as $\left[ {w_{\min } ,w_{\max } } \right] = \left[ {0.8,1.2} \right]$, and the other parameters were taken as the default values in Matlab. Figure 13 shows that there are $6 \times 13 + 13 + 13 \times 4 + 4 + 4 \times 4 + 4 = 167$ weights and thresholds to be optimized, so the particle swarm dimension *M* was set to 167.

**Figure 14:**
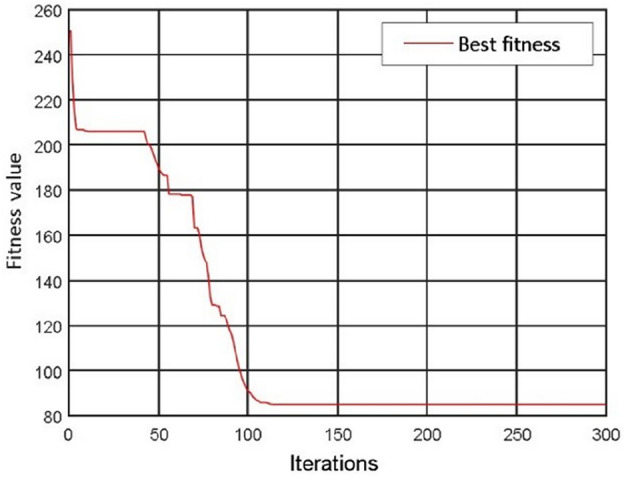
Optimum fitness curve of the PSO algorithm.

**Figure 15:**
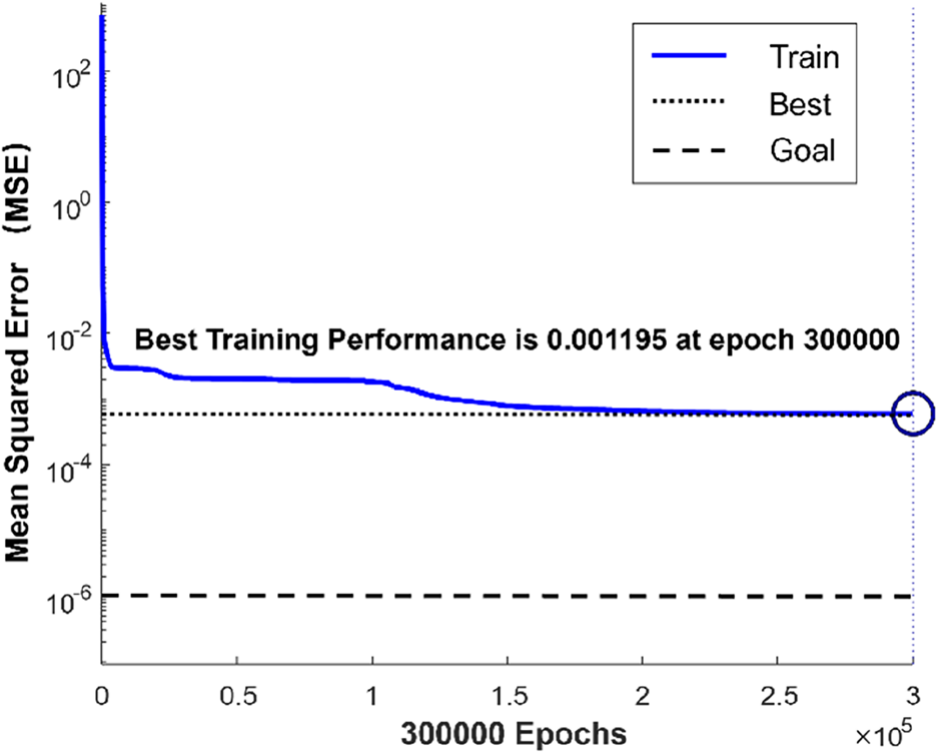
Variation of the mean square error during the BP network training.

The optimum fitness curve^54,55^ of the PSO algorithm is shown in Fig. 14. The best fitness was found after the population had been iterated about 110 times. The particle position of the best fitness was used as the initial weight and threshold of the BP neural network, and then the network training was carried out. The variation of the mean square error during the training is shown in Fig. 15. It shows that the network training finished when the maximum number of network training is 300,000, and the mean square error is less than 0.0012.

### Back analysis of surrounding rock parameters

The trained PSO-BP network was further used to inversely analyze the surrounding rock parameters of the Shuangjiangkou underground powerhouse. To verify the advantages of the PSO-BP, both the PSO-BP and the BP methods were used to inversely analyze the parameters of the surrounding rock. The network structures of the two methods are consistent. The measured displacement increments of six monitoring points listed in Table 2 were used as the input data of these two network, and the output surrounding rock parameters are listed in Table 5.

**Table 5:** Back-analyzed surrounding rock parameters of the Shuangjiangkou underground powerhouse.

Method	Modulus *E*/GPa	Poisson 's ratio *μ*	Internal friction angle *φ*/°	Cohesion *c*/MPa
BP	34.95	0.25	50.98	0.91
PSO-BP	36.4	0.26	58.2	1.48

**Figure 16:**
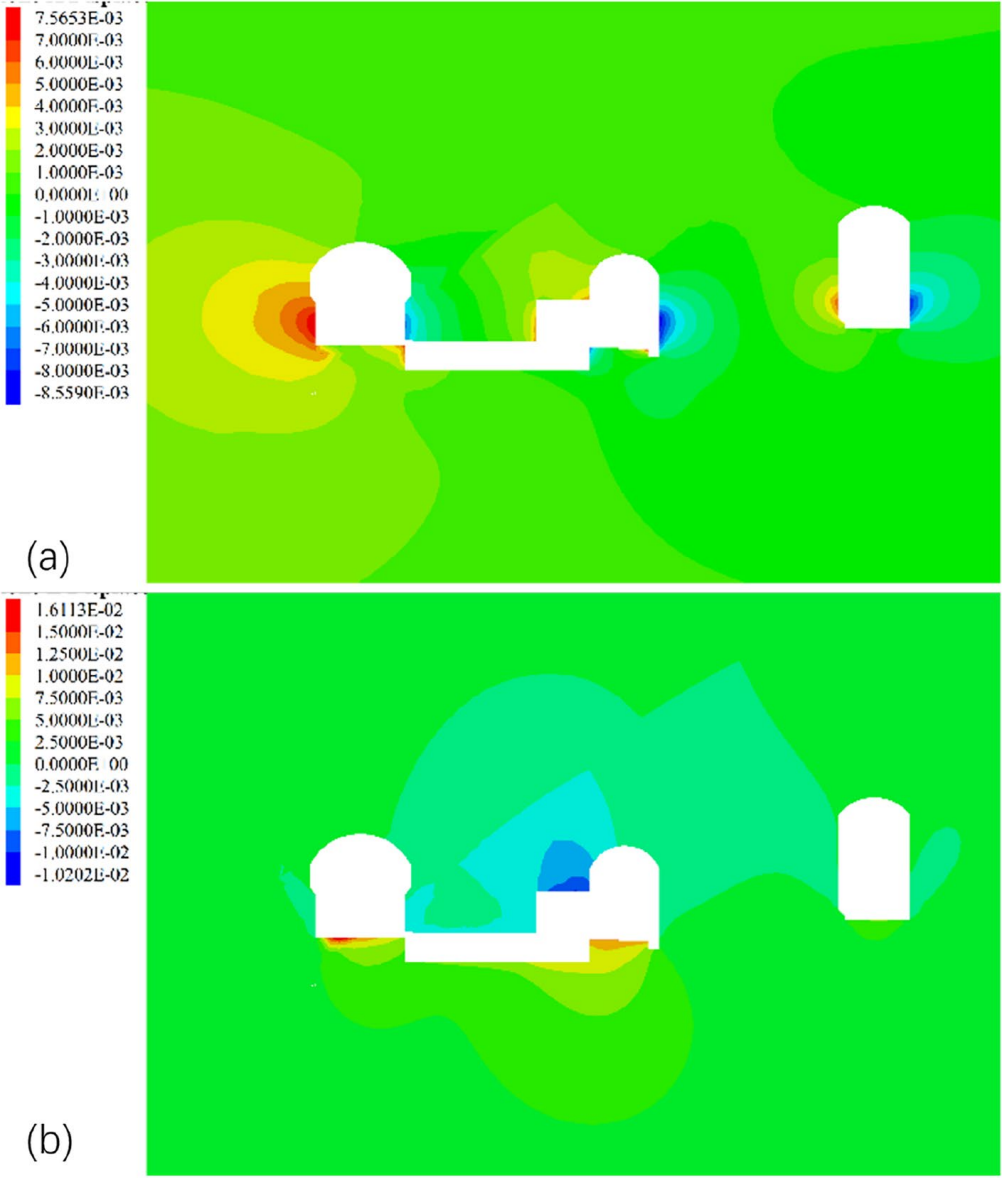
Displacement increment distribution of surrounding rock in the section of 3# electric generator during the fourth stage layer of excavation (unit: m). (**a**) horizontal displacement increment and (**b**) vertical displacement increment.

The back-analyzed parameters of surrounding rock in Table 5 were substituted into the numerical model to simulate the fourth layer of excavation of the underground powerhouse, and the displacement increments of surrounding rock in the fourth layer of excavation was obtained. Figure 16 shows the distribution of displacement increments calculated by the PSO-BP method in the section of 3# electric generator. It shows that the deformation increment caused by the fourth layer of excavation mainly occurs in the newly exposed surrounding rock of the excavation, and the surrounding rock deforms to the free surface of the cavern. The maximum horizontal displacement increment is 7.6 mm and occurs on the upstream side wall of the main powerhouse, and the maximum horizontal displacement increment on the downstream side wall is about 6 mm. The deformation increment of the surrounding rock at the vault is small, so the fourth layer of excavation has little effect on its deformation.

**Figure 17:**
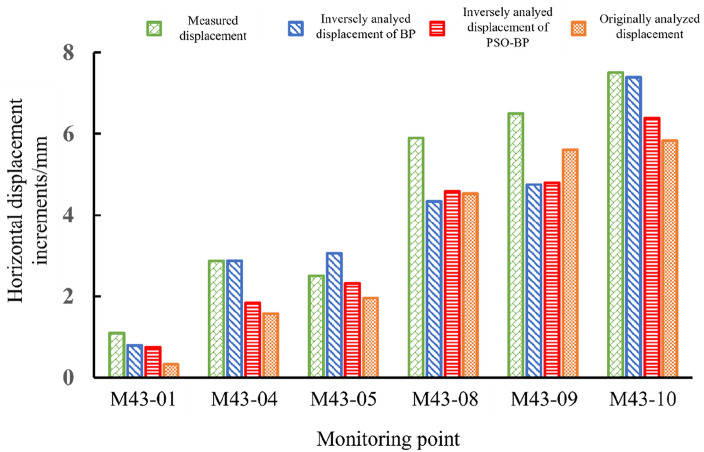
Comparison of the measured, originally analyzed and inversely analyzed horizontal displacement increments of six monitoring points in the section of 3# electric generator.

Figure 17 shows the measured, originally analyzed and inversely analyzed horizontal displacement increments of six monitoring points in the section of 3# electric generator. The originally analyzed displacement increments with the initial mechanical parameters are much larger than the measured displacement increments, and the inversely analyzed displacement increments are closer to the measured displacement increments. Table 6 lists the displacement increments calculated by two methods. The mean squared error between the displacement increment obtained by the BP method and the measured value is 1.05, and the mean relative error is 54.07%. The mean squared error between the displacement increment obtained by the PSO-BP method and the measured value is 0.22, and the mean relative error is 31.25%. Thus, the back-analyzed parameters obtained by the PSO-BP method reflect the mechanical properties of the surrounding rock better than the parameters obtained by the BP method.

**Table 6:** Comparison of measured displacement increments and calculated displacement increments based on BP and PSO-BP methods (unit: mm).

Method	M43-01	M43-04	M43-05	M43-08	M43-09	M43-10	Mean squared error	Mean relative error
Measurement	0.33	1.57	1.96	4.53	5.61	5.83	–	–
BP	0.79	2.87	3.06	4.34	4.76	7.39	1.05	54.07%
PSO-BP	0.75	1.84	2.31	4.59	4.80	6.38	0.22	31.25%

### Accuracy evaluation of the inversion analysis

In this paper, the posterior variance method^56^ was used to objectively evaluate the accuracy of the PSO-BP inversion analysis. The displacement increment is used to evaluate the inversion results. The evaluation process is as follows^57,58^.

1. Displacement increment residual:
  5 $$ \varepsilon (i) = x(i) - \hat{x}(i),\quad i = 1,2, \ldots ,n $$
  where *ε*(*i*) is the residual error of displacement increment of the measuring point *i*; *x*(*i*) is the measured displacement increment of the measuring point *i*; $\hat{x}(i)$ is the numerically calculated displacement increment of the measuring point *i*; *n* is the number of measuring points.
2. Mean of the displacement increment residuals:
  6 $$ \overline{\varepsilon } = \frac{1}{n}\mathop \sum \limits_{i = 1}^{n} \varepsilon (i) $$
  Mean square error of the displacement increment residuals:
  7 $$ S_{1}^{2} = \frac{1}{n}\mathop \sum \limits_{i = 1}^{n} (\varepsilon (i) - \overline{\varepsilon })^{2} $$
3. Mean of the measured displacements:
  8 $$ \overline{x} = \frac{1}{n}\mathop \sum \limits_{i = 1}^{n} x(i) $$
  Mean square error of the measured displacement:
  9 $$ S_{2}^{2} = \frac{1}{n}\mathop \sum \limits_{i = 1}^{n} (x(i) - \overline{x})^{2} $$
4. The indexes of the posterior variance method:
  10 $$ C = \frac{{S_{1} }}{{S_{2} }} $$
  11 $$ P = P\left\{ {|\varepsilon (i) - \overline{\varepsilon }| < 0.6745S_{2} } \right\} $$
  where *C* is the posterior error ratio; *P* is the probability of a small error. According to these two indexes of the posterior variance method, the evaluation grades^57,58^ are classified into four levels as shown in Table 7.

**Table 7:** Evaluation grades based on the indexes of the posterior variance method.

Posterior error ratio *C*	Small error probability *P*	Evaluation grades
$C \le 0.35$	$P \ge 0.95$	Excellent
$0.35 < C \le 0.50$	$0.80 \le P < 0.95$	Good
$0.50 < C \le 0.65$	$0.70 \le P < 0.80$	Qualified
$C > 0.65$	$P < 0.70$	Unqualified

The measured and inversely analyzed displacement increments were further analyzed by the posterior variance method. The analysis results including residuals, mean square error of residuals and displacement increments, and the indexes of the posterior variance method are shown in Table 8. The posterior error ratio *C* = 0.045 is smaller than 0.35 and indicates the evaluation grade is excellent. The probability of small error *P* = 0.999 is larger than 0.95 and also indicates the evaluation grade is excellent. Therefore, the overall evaluation grade of the back analysis is excellent.

**Table 8:** The evaluation grade of the back analysis based on the posterior variance method.

Measuring point number	Inversely analyzed displacement increments/mm	Measured displacement increments/mm	Residual *ε*	Mean square error of residuals $S_{1}^{2}$	Mean square error of displacement increments $S_{2}^{2}$	*C*	*P*
M43-01	0.75	0.33	− 0.42				
M43-04	1.84	1.57	− 0.27				
M43-05	2.31	1.96	− 0.35	0.21	4.48	0.045	0.999
M43-08	4.59	4.53	− 0.07				
M63-09	4.80	5.61	0.81				
M43-10	6.38	5.83	− 0.57				

## Conclusion

The BP neural network is sensitive to the initial parameters, so the global optimization ability of the PSO is used to optimize the initial weights and thresholds of the BP neural network. The PSO-BP algorithm and the cloud computing technology were integrated into an intelligent feedback analysis cloud program for underground engineering safety monitoring to facilitate real-time feedback analysis of surrounding rock parameters. The program was constructed with a web platform and a cloud server. The web platform was designed as simple as possible to make user easily input some necessary and basic parameters, and all the professional software were called by the program to automatically run the simulations in the could server. The program provides a practical path for users to conveniently, quickly, and intelligently carry out numerical simulations of an underground engineering and feedback analysis of surrounding rock parameters.

The cloud program was further applied to the feedback analysis of the surrounding rock parameters of the Shuangjiangkou underground powerhouse. The elastoplastic mechanical parameters of surrounding rock were inversely obtained based on the measured displacement increments during the fourth layer of excavation. By comparing the originally and inversely simulated displacement increments with the measured displacement increments, the originally simulated displacement increments are much larger than the measured values, and the inversely simulated displacement increments are closer to the measured values. The analysis results of the posterior variance method show that the posterior error ratio of the inversely simulated displacement increments is *C* = 0.045, and the probability of small error is *P* = 0.999. These two indexes of the posterior variance method demonstrate the evaluation grade of the back analysis is excellent, so the intelligent feedback analysis cloud program is feasible in practical engineering application.

## Data Availability

The datasets used in the current study are available from the corresponding author upon reasonable request.
